# The effect of alexithymia on depression: evidence from meta-analysis

**DOI:** 10.3389/fpsyg.2025.1465286

**Published:** 2025-06-02

**Authors:** Zhijun Liu, Shiwei He, Chunna Hou

**Affiliations:** ^1^Department of Sociology, Changchun University of Science and Technology, Changchun, China; ^2^School of Psychology, Northeast Normal University, Changchun, China

**Keywords:** alexithymia, depression, meta-analysis, difficulty in describing feelings, difficulty in identifying feelings, externally oriented thinking

## Abstract

**Introduction:**

Depression is primarily characterized by persistent low mood and cognitive dysfunction, whereas whereas alexithymia refers to difficulties in cognitively processing emotions. Although alexithymia being recognized as a risk factor for depression, there is no clear scholarly consensus on its exact role.

**Methods:**

This investigation employs a meta-analytic random effects model to examine the relationship between alexithymia and depression. The analysis draws on data from 35 studies involving 23,085 individuals.

**Results:**

Several key findings are revealed: Firstly, the total alexithymia score shows a positive correlation with depression severity, possibly influenced by cultural differences between Eastern and Western populations. Secondly, Difficulty in Describing Feelings (DDF) and Difficulty in Identifying Feelings (DIF) are significantly associated with depression, while Externally Oriented Thinking (EOT) has a weaker link. Thirdly, the relationship varies across different demographic groups and depends on the depression assessment tool used.

**Discussion:**

These results emphasize the importance of examining not only the overall relationship between alexithymia and depression but also its specific dimensions. Additionally, the study explores the rational explanation of the interplay between alexithymia and depression within a Chinese cultural context and within the framework of the interpersonal theory of depression.

## 1 Introduction

Depression, from a medical standpoint, is a debilitating mental and physical condition characterized by persistent low mood and cognitive dysfunction. It profoundly affects an individual’s social functioning and ranks among the most common mental disorders (Rock et al., 2014). The recurrence rate of depressive disorders is notably high, ranging from 50 to 85%, with relapses occurring within 2 years of initial onset. The lifetime prevalence rate of depressive disorders among adults is approximately 6.8%. In addition to severe social impairments, depression imposes a significant economic burden (Trautmann and Wittchen, 2016; Tran et al., 2018). Global estimates suggest that treatment costs could reach nearly US$16 trillion by 2030, positioning depression as the fourth highest global burden of disease, as recognized by the World Health Organization (Bloom et al., 2011; Patel et al., 2018).

Alexithymia, often described as “inability to express emotions” or “affective dysphoria” (Sifneos, 1973), refers to an individual’s difficulty in recognizing and expressing their own or others’ emotions. It reflects deficiencies in the cognitive processing of emotions, impairing social–emotional communication, such as empathy, and leading to impaired social functioning (Wingbermühle et al., 2012). The Toronto Alexithymia Scale (TAS Scale), a widely used self-reported assessment tool, is commonly employed for its evaluation (Fujiwara, 2018). Alexithymia, considered a stable personality trait, is characterized by difficulty in identifying personal emotions (Difficulty in Identifying Feelings - DIF), differentiating emotions from somatic sensations (Difficulty in Describing Feelings - DDF), and displaying limited emotional imagination alongside an externally oriented cognitive style (Externally Oriented Thinking - EOT) (Taylor et al., 1999; Kooiman et al., 2002). Research shows that individuals from Eastern collectivist cultures may experience greater challenges with alexithymia. Unlike Western individualistic cultures (IDV), Eastern collectivist cultures (COL;see Gelfand et al., 2004; Hofstede, 2011) are rooted in Confucian values, which place a strong emphasis on emotional regulation (Butler et al., 2007; Dere et al., 2013). Deficits in emotional regulation play a key role in this issue (Marroquín, 2011).

Scholars have emphasized the need to investigate risk factors or determinants influencing depression, as this can inform preventive strategies aimed at reducing the likelihood of developing such mental disorders, thereby enhancing public mental health (Guan et al., 2022). One high-risk factor receiving increased attention is alexithymia (Hou et al., 2024); however, the current understanding of the relationship between them remains subject to debate.

### 1.1 The relationship between alexithymia and depression

Some scholars advocate that alexithymia influences depression, positing it as susceptible to and potentially influential in the development of depression (Van Der Cruijsen et al., 2019). Numerous studies have highlighted a potential correlation between total alexithymia scores and depression (Kenangil et al., 2020; Farhoumandi et al., 2022; Shi et al., 2022; Koppelberg et al., 2023), suggesting alexithymia as a predisposing factor for psychiatric disorders, particularly depression (Kinnaird et al., 2019). Meta-analytic evidence from Li et al. (2015) further supports this assertion, revealing a moderate correlation (*r* = 0.459) between total alexithymia scores and depression severity across a sample of 3,572 subjects from 19 Western studies.

Conversely, some scholars contest the association between alexithymia and depression. Some studies find that the two failed to establish a significant correlation in smokers and extremely obese individuals (Grabowska et al., 2005; Da Ros et al., 2011), while others report correlation coefficients below Cohen’s (1988) threshold for meaningfulness (Reker et al., 2010; Luca et al., 2013). Despite efforts to elucidate the relationship, dissenting views persist. Barbasio et al. (2015) found no significant correlation between alexithymia, depression, and illness perception among 100 patients with Systemic Lupus Erythematosus (SLE). Recent research in the autism community also indicates no impact of alexithymia on depression (Oakley et al., 2022).

Given the notable challenges associated with alexithymia in East Asian cultures, the present study examines both Western and Eastern cultural contexts. Numerous theorists have argued that the two cultures exhibit distinct differences in cultural beliefs, norms, and values, which may influence individuals’ abilities to identify and communicate emotions (e.g., Markus and Kitayama, 1994). Building on this foundational research, the current study proposes *H1*: There is a significant correlation between total alexithymia scores and depression, with potential differences in the correlation between Eastern and Western cultural settings.

### 1.2 There are differences in the effects of alexithymia factors on depression

While scholars recognize alexithymia as a risk factor for depression, considerable debate persists regarding the specific effects of its constituent factors. Difficulty Identifying Feelings (DIF) Factor: Empirical evidence, including a follow-up study involving 4,431 individuals from Northern Finland, highlights DIF as notably associated with depression (Ramzi et al., 2023). De Berardis et al. (2008) asserted DIF as the primary predictor of depression based on a study of medication-naive patients with Major Depression (MD). Similarly, in Korean depressed patients, measures of alexithymia and stress response patterns corroborated the significant correlation between DIF and depression (Kim et al., 2009). Nonetheless, conflicting findings emerge, as some studies fail to establish a significant correlation between DIF and depression (He et al., 2022).

Difficulty Describing Feelings (DDF) Factor: In contrast to De Berardis et al. (2008) and Leweke et al. (2011) found a significant correlation between the DDF factor and depression. Intriguingly, while DIF and Externally Oriented Thinking (EOT) were not correlated with depression, DDF emerged as a potential discriminatory feature of depression. Externally Oriented Thinking (EOT) Factor: Although several studies report no correlation between EOT and depression severity (Marchesi et al., 2008; Bamonti et al., 2010; Son et al., 2012), Saarijärvi et al. (2006) observed unaltered EOT scores during a longitudinal study of outpatients with Major Depressive Disorder. However, recent evidence suggests a significant positive association between EOT and depression in cancer patients (Ozonder Unal and Ordu, 2023).

In light of these findings, this study proposes *H2*: alexithymia and depression may exhibit inconsistencies, with weakly correlated or uncorrelated alexithymia factors potentially existing.

### 1.3 Impact of additional variables on the alexithymia-depression relationship

Furthermore, demographic variables such as subject group, gender, depression measurement tools, and publication age of literature may influence the correlation between alexithymia and depression.

#### 1.3.1 Subject group

The prevalence of alexithymia varies across community and clinical samples, highlighting the role of subject group in its association with depression. Populations with psychological or somatic disorders, such as autism, Parkinson’s disease, and sleep disorders, show particularly high rates of alexithymia. Notably, the incidence of alexithymia may reach up to 55% among autistic adolescents (Milosavljevic et al., 2016). This suggests that the elevated prevalence of alexithymia in these groups may impact the extent to which alexithymia predicts depression. Hence, this study posits *H3*: Different subject groups affect the conclusion regarding the correlation between alexithymia and depression.

#### 1.3.2 Gender factors

Gender plays a complex role in alexithymia and its association with depression. While some studies suggest higher mean scores of alexithymia in males, others dispute gender differences. Nonetheless, the female gender is prominently associated with depression due to various biological, psychological, and social factors. Therefore, this study proposes *H4*: Gender factors influence the association between alexithymia and depression.

#### 1.3.3 Depression measurement tools

Inconsistencies in the alexithymia-depression relationship may stem from differences in depression measurement tools. Given the variety of tools employed in academia, including self-report measures and clinician-administered scales, the choice of tool may affect the observed correlation (see Table 1).

**Table 1:** Depression measurement tools involved in literature over calendar years.

Scale name	The establishment of people	years
BDI	Back	1967
CES-D	Sirodff	1977
HAMD	Hamilton	1960
SDS	Zung	1965

BDI, Beck depression Inventory; HAMD, Hamilton Depression Scale; SDS, self-rating depression scale; CSE-D, center for epidemiologic studies depression scale.

Although all these scales have passed the measurement methodology test, there are differences in the applicable groups, assessment methods, and scoring methods, which may impact the study results. Firstly, the Hamilton Depression Rating Scale (HAMD) was utilized in patients with depression to evaluate illness severity, while the remaining instruments were commonly employed in the general population to screen for symptoms. Secondly, the Beck Depression Inventory (BDI), Center for Epidemiologic Studies Depression Scale (CES-D), and Self-Rating Depression Scale (SDS) are self-report measures, whereas the HAMD is an observer-rated measure administered by trained personnel. Thirdly, regarding the scoring method: the HAMD employs a 3- or 5-point scale based on severity, while the other scales use 4-point scales, albeit with subtle variations. For instance, the BDI assesses severity, while the SDS and CES-D measure time-frequency. Additionally, considering that the literature in the meta-analysis encompasses both Chinese and foreign languages, equivalence across five systematic assessment aspects—content, semantics, methodology, norms, and constructs—must be considered in cross-cultural research, as recommended by Flaherty et al. (1988).

Consequently, this study posits *H5*: Different depression measurement tools may influence the correlation between alexithymia and depression.

#### 1.3.4 Publication year

It may also potentially affect the correlation between depression and alexithymia. Prior studies have affirmed the increasing prevalence of depression in the general population, partly attributed to evolving societal dynamics that exacerbate stressors leading to depression. Conversely, heightened societal awareness of mental illnesses, including depression, has fostered open discussions in recent years, thereby enhancing recognition and acknowledgment of depression. However, it is noteworthy that literature also indicates stable depression trends, underscoring relevant changes in depression over time.

Thus, this measure of publication year tests the hypothesis that the correlation between alexithymia and depression has changed over time, with the expectation of *H6* that more recent studies would find a stronger correlation.

## 2 Methods

### 2.1 Literature search and selection

Sterne et al. (2011) emphasized the pivotal role of a systematic and exhaustive literature search preceding meta-analysis in mitigating publication bias, advocating for proactive measures over *post hoc* analyses. In line with this principle, our study meticulously scrutinized both Chinese and English literature spanning the East and West over a continuous period of 22 years (2001–2023). For English sources, we utilized Web of Science, PubMed, and Google Scholar, employing the search terms “Alexithymia” and “depression.” Additionally, to enhance our understanding of the evolving relationship between alexithymia and depression, we incorporated longitudinal studies specifically focusing on “follow-up duration,” “longitudinal.” Chinese databases such as China National Knowledge Infrastructure, China Science and Technology Journal Database, and Wan-fang database were queried using equivalent Chinese terms.

**Figure 1:**
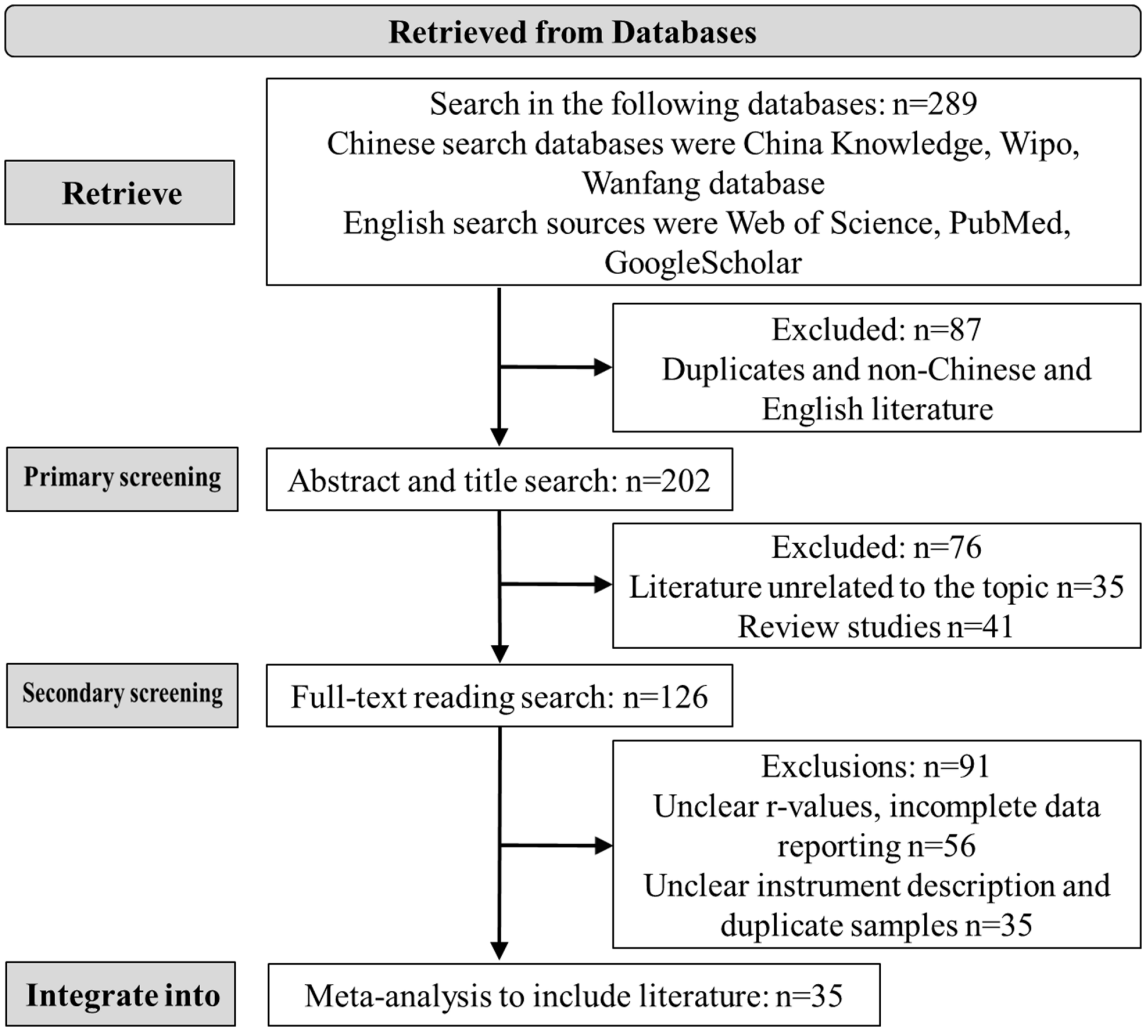
The flowchart illustrating the literature screening process.

Following the PRISMA statement guidelines (Moher et al., 2009), we meticulously screened the identified literature based on the following criteria: (1) inclusion of quantitative empirical studies elucidating the correlation between alexithymia and depression, (2) provision of descriptions regarding the instruments employed to assess alexithymia and depression, (3) independence of studies, excluding those with overlapping samples or duplications, (4) exclusion of studies deemed low quality (scoring 0–3 points), and (5) clarity on sample sizes. To ensure comprehensiveness, we conducted a secondary search within the references of retrieved articles (see Figure.1).

### 2.2 Literature quality assessment and coding

The Meta-Analysis Assessment Scale, as devised by Boyle (1998), was employed for systematic evaluation, encompassing sample representativeness, measurement tool validity, and analytical results across eight criteria. Each assessor independently conducted the assessment, assigning scores ranging from 0 to 8, wherein scores falling within 0 to 3 denoted low quality, 4 to 6 indicated moderate quality, and a score of 7 or higher signified high quality. The inter-rater agreement, measured by the Kappa coefficient, yielded a value of 0.84, suggesting substantial concordance between assessors, as per Orwin and Vevea’s (2009) classification, where values exceeding 0.75 denote very good agreement (specifically: 0.40 to 0.59 for fair, 0.60 to 0.74 for fairly good, and 0.75 and above for very good agreement). This study encompasses 35 investigations, with sample sizes ranging from 40 to 10,908, totaling 23,085 participants, of whom female representation exceeds 53.2%. The descriptive information for each study is shown in Table 2.

**Table 2:** Included the original studies in the meta-analysis.

No.	Author (year)	*r*	Sample size	Male ration	district	Subjects status	Test tools	
							Depression	Alexithymia
1	Honkalampi et al. (2001)	0.506	1888	0.448	West	Barrier-free	BDI	TAS-20
2	Lu et al. (2002)	0.640	87	0.391	East	Mental disorder	HAMD	TAS-26
3	Müller et al. (2003)	0.370	174	0.391	West	Barrier-free	SDS	TAS-20
4	Sayar et al. (2003)	0.370	100	0.280	West	Physical impairment	HAMD	TAS-20
5	De Gennaro et al. (2004)	0.439	554	0.134	West	Barrier-free	CES-D	TAS-20
6	Eizaguirre et al. (2004)	0.511	151	0.000	West	Mental disorder	BDI	TAS-20
7	Motan and Gençöz (2007)	0.300	145	0.179	West	Barrier-free	BDI	TAS-20
8	De Berardis et al. (2008)	0.440	145	0.476	West	Mental disorder	HAMD	TAS-20
9	Bratis et al. (2009)	0.514	95	0.179	West	Barrier-free	BDI	TAS-20
10	Bamonti et al. (2010)	0.410	134	0.400	West	Barrier-free	BDI-II	TAS-20
11	Herbert et al. (2011)	0.390	155	0.568	West	Barrier-free	BDI-II	TAS-20
12	Luca et al. (2013)	0.290	75	0.333	West	Mental disorder	HAMD	TAS-20
13	Yalınay Dikmen et al. (2020)	0.485	145	0.234	West	Physical impairment	BDI	TAS-20
14	Liu and Wang (2019)	0.420	10,908	0.510	East	Barrier-free	SDS	TAS-20
15	Dong et al. (2019)	0.349	119	0.613	East	Mental disorder	BDI	TAS-26
16	Kim et al. (2020)	0.610	86	0.686	East	Mental disorder	BDI	TAS-20
17	Kenangil et al. (2020)	0.280	42	0.570	West	Physical impairment	BDI	TAS-20
18	Pei et al. (2021)	0.478	413	0.107	East	Barrier-free	BDI-II	TAS-20
19	Tan et al. (2021)	0.440	365	1.000	East	Barrier-free	BDI-II	TAS-20
20	Tang et al. (2022)	0.500	817	0.455	East	Barrier-free	CES-D	TAS-20
21	Ren et al. (2022)	0.650	511	0.431	East	Barrier-free	CES-D	TAS-20
22	Chen et al. (2022)	0.420	456	1.000	East	Barrier-free	BDI	TAS-20
23	Miao et al. (2022)	0.550	394	0.373	East	Mental disorder	CES-D	TAS-20
24	Farhoumandi et al. (2022)	0.418	334	0.476	West	Barrier-free	BDI-II	TAS-20
25	Shi et al. (2022)	0.389	183	0.563	East	Physical impairment	SDS	TAS-20
26	Oakley et al. (2022)	0.220	179	0.693	West	Mental disorder	BDI	TAS-20
27	Zhang B. et al. (2023)	0.604	2,343	0.221	East	Mental disorder	Other	TAS-20
28	Zhang H. et al. (2023)	0.342	842	0.720	East	Physical impairment	Other	TAS-20
29	Xiong et al. (2023)	0.650	392	0.232	East	Mental disorder	BDI-II	TAS-20
30	Lv et al. (2023)	0.337	86	0.000	East	Mental disorder	Other	TAS-20
31	Lv et al. (2023)	0.391	40	1.000	East	Mental disorder	Other	TAS-20
32	Oakley et al. (2022)	0.550	337	0.674	West	Barrier-free	BDI-II	TAS-20
33	Oakley et al. (2022)	0.340	135	0.622	West	Barrier-free	BDI-II	TAS-20
34	Oakley et al. (2022)	0.490	179	0.693	West	Mental disorder	BDI-II	TAS-20
35	Oakley et al. (2022)	0.210	76	0.671	West	Mental disorder	BDI-II	TAS-20

### 2.3 Data processing

This study utilized Pearson’s product-difference correlation coefficient (r) between alexithymia and depression as the effect size indicator, favored for its advantages over alternative measures (Rosenthal and DiMatteo, 2001). Potential publication bias was evaluated using funnel plots in CMA 3.3 (Comprehensive Meta-Analysis Version 3.3) and the Egger regression test. Specifically, a symmetrical funnel plot suggests an absence of publication bias, while non-significant results from the Egger regression imply negligible bias (Egger and Smith, 1998; Viechtbauer, 2007).

Furthermore, Cochran’s Q test with the I2 statistic was employed to gauge heterogeneity among studies, where *I^2^ = 100% × (Q-df)/Q* (df representing degrees of freedom). As per Higgins et al. (2003), an *I^2^* value exceeding 75% indicates substantial heterogeneity, justifying the use of a random effects model for meta-analysis. Given the inclusion of diverse subject groups from both Eastern and Western contexts, and in line with Quintana’s (2015) recommendation, the random effects model was adopted.

Additionally, this study conducted subgroup analyses and meta-regression to explore potential moderators influencing heterogeneity. These analytical methods, deemed indispensable for meta-analyses, were employed to investigate factors contributing to variation (Lee et al., 2022).

Following Card’s (2012) criteria, each moderator variable required a minimum of five representative effect sizes for analysis. Finally, sensitivity analyses were performed to ensure the robustness of the meta-analytic findings, mitigating the influence of small sample size studies or literature not entirely meeting the screening criteria (Li et al., 2018).

## 3 Results

### 3.1 Publication Bias and heterogeneity test

**Figure 2:**
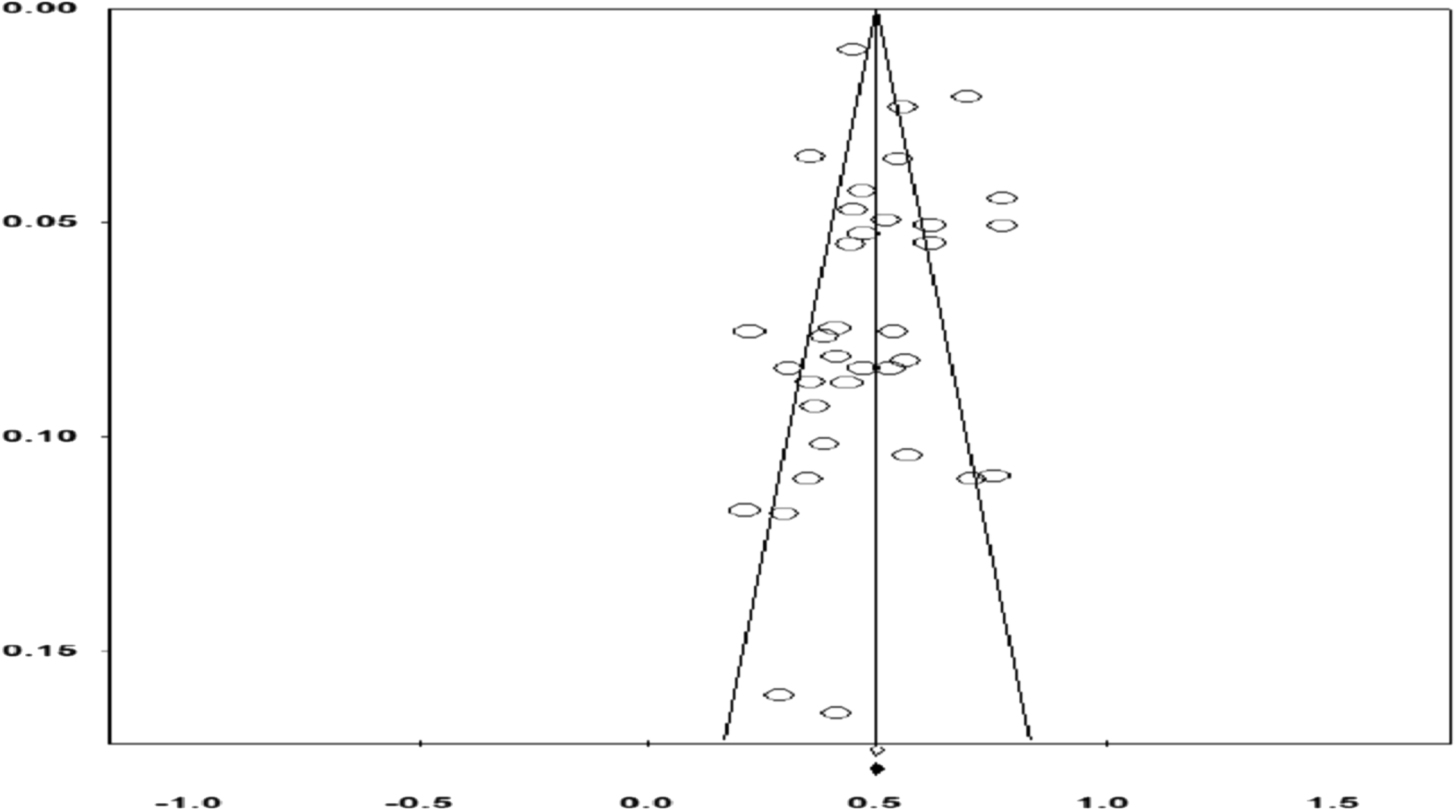
A funnel plot of publication bias.

Firstly, examination of the funnel plot revealed a concentration of effect sizes in the upper-middle section, evenly distributed on either side of the overall effect, forming a symmetrical funnel shape (Figure 2). Subsequently, Egger’s test indicated no statistically significant publication bias, yielding *β* = 0.045, *p* = 0.951, with a 95% CI = [−1.441, 1.532]. Following guidelines by Higgins et al. (2019), no evidence of publication bias was observed.

Secondly, to assess heterogeneity among included effect sizes and validate the selection of the random effects model, this study conducted a comprehensive analysis. The results demonstrated a significant departure from the null hypothesis (*Z* = 75.933, *p* < 0.001), alongside Cochrane’s Q statistic of 283.226 with 34 degrees of freedom, yielding *p < 0.001*, and an *I*^2^ value of 87.995%, exceeding the threshold of 75% proposed by Higgins et al. (2003). This substantial level of heterogeneity indicates that variations in effect sizes concerning the relationship between alexithymia and depression across the literature are not merely attributed to chance but likely reflect genuine underlying factors. Consequently, employing a random effects model is deemed appropriate, necessitating subsequent meta-regression and subgroup analyses to delve deeper into potential contributors to study heterogeneity.

### 3.2 Correlation test between alexithymia TAS Total score and depression

The Project 35 study revealed a significant association between total TAS scores for alexithymia and depression, encompassing 23,085 participants. Employing a random-effects model, the overall effect size was *r* = 0.455, with a *95% CI =* [0.417, 0.491], across 35 studies. Individual study effect sizes ranged from 0.313 to 0.462, all indicating positive correlations. Sensitivity analyses, excluding the smallest sample size studies (*n* = 40), resulted in an adjusted overall effect size of *r =* 0.456, *95% CI* = [0.417, 0.492], across 34 studies, indicating a stable association. Cohen’s (1988) conventions classify correlations of *r =* 0.10, *r =* 0.30, and *r* = 0.50 as weak, moderate, and high, respectively. In this study, the association between alexithymia TAS total scores and depression ranged from moderate to high correlations. Notably, Gignac and Szodorai (2016) suggested that an *r* = 0.30 could be considered highly correlated in meta-analytic contexts, thereby characterizing the present study’s findings as demonstrating a highly positive correlation between total TAS score of alexithymia and depression. Additionally, sensitivity analyses excluding either sample yielded consistent results, suggesting a stable association.

Furthermore, the study investigated the influence of cultural background factors through subgroup analyses. Firstly, employing a random effects model for Eastern cultural background revealed an overall effect size of *r =* 0.498, *95% CI* = [0.438, 0.553], across 16 studies, with significant heterogeneity observed (*Q* = 231.949, *df* = 15, *p* < 0.001; *I^2^* = 93.533%). Sensitivity analyses, excluding studies with the smallest sample size, yielded an adjusted overall effect size of *r* = 0.501, *95% CI* = [0.440, 0.557], across 15 studies, indicating a slight increase in association. Secondly, the random effects model for Western cultural background yielded an overall effect size of *r =* 0.416, *95% CI* = [0.371, 0.460], across 19 studies, with indications of substantial heterogeneity (*Q* = 50.384, *df* = 18, *p* = 0.001; *I^2^* = 64.274%). Sensitivity analyses, excluding the smallest sample size study, yielded a consistent overall effect size of *r =* 0.406, *95% CI* = [0.359, 0.450], across 18 studies. Notably, confidence intervals for overall effect sizes varied considerably between cultural contexts, suggesting differing correlations between alexithymia and depression in Eastern and Western cultures, with a potentially higher correlation observed in Eastern cultures.

### 3.3 Correlation test between alexithymia sub-dimensions and depression

To investigate whether TAS subscales contribute to heterogeneity in the alexithymia-depression association, subgroup analyses of TAS subscales were conducted involving 7,885 participants across 30 studies (see Supplementary material). Visual inspection of funnel plots revealed symmetrical distributions, and Egger’s test indicated non-significant publication bias [*β* = 1.617, *p* = 0.117, *95% CI* = (−0.430, 3.665)]. Firstly, random effects model results for DDF subscale subgroup analysis exhibited a moderate correlation, with a mean effect size of *r* = 0.331, *95% CI* = [0.268, 0.391], across 30 studies. Significant inter-study heterogeneity was observed (*Q* = 232.862, *df* = 29, *p* < 0.001; *I^2^* = 87.546%). Secondly, subgroup analysis focusing on DIF subscales showed a mean effect size of *r* = 0.411, 95*% CI* = [0.357, 0.463], across 30 studies, indicating a moderate correlation. However, substantial and significant inter-study heterogeneity was noted (*Q* = 194.679, *df* = 29, *p* < 0.001; *I^2^* = 85.104%).

Conversely, EOT subscale analysis revealed a weak correlation with depression, with a mean effect size of *r* = 0.120, *95% CI* = [0.080, 0.159], across 30 studies. Heterogeneity between studies was evident (*Q* = 69.015, *df* = 29, *p* < 0.001; *I^2^* = 57.980%). Thus, differences were observed in the associations between TAS subscales and depression. While DDF and DIF showed moderate correlations, EOT exhibited a weak correlation, suggesting a minimal contribution. This finding supports H2.

Moreover, this study employed meta-analytic structural equation modeling to explore TAS subscales’ role in the alexithymia-depression association. A correlation matrix for structural equation modeling was derived from 14 studies providing correlation coefficients between DDF, DIF, EOT, and depression. A reconciled mean was utilized to obtain observations for the structural variance model (*N* = 245.388) (see Table 3).

**Table 3:** The correlation matrix between the alexithymia subscale and depression.

Dimension	DIF	DDF	EOT	Depression
DIF	1			
DDF	0.429	1		
EOT	0.351	0.204	1	
Depression	0.411	0.331	0.120	1

**Figure 3:**
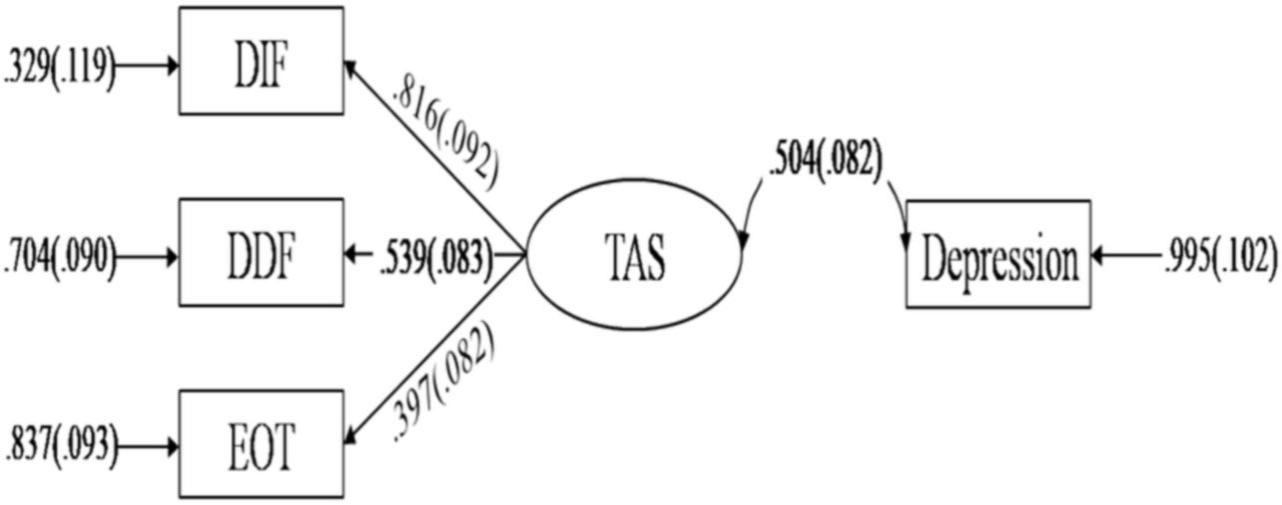
Meta-analytical structural equation model for the relationship between TAS subscales and depression.

The results of the model fit showed a moderate fit with *χ2* = 3.820, *df* = 2, *CFI* = 0.982, *TLI* = 0.946, *RMSEA* = 0.069 [*90% CI* = 0.000, 0.186], *SRMR* = 0.033. The results indicated a significant association between the alexithymia latent variable and depression (*ρ* = 0.504*, p* < 0.001), providing supporting evidence for the association between alexithymia and depression. The factor loadings for DIF, DDF, and EOT were all significant (*ps* < 0.001), but EOT explained the least amount of variance (*R^2^* = 0.158), less than 20%, compared to DIF (*R^2^* = 0.670) and DDF (*R^2^* = 0.293), which explained more than 25% of the variance. This result once again highlights the relatively weak contribution of EOT (see Figure 3).

### 3.4 Moderating effects test

To explore potential moderator variables affecting the relationship between alexithymia and depression, subgroup analyses and meta-regression were conducted. Subgroup analyses investigated the influence of group type and heterogeneity of measurement instruments. Notably, significant between-study heterogeneity by group type was observed (*Q* = 9.999, *df* = 2, *p* = 0.007), supporting Hypothesis 3. For the mental disorders group, an overall effect size of *r* = 0.470, *95% CI* = [0.393, 0.540], across 15 studies, exhibited substantial heterogeneity (*Q* = 95.533, *df* = 14, *p* < 0.001; *I^2^* = 85.345%). Similarly, the no-impairment group showed an overall effect size of *r* = 0.461, *95% CI* = [0.419, 0.501], across 15 studies, with significant heterogeneity (*Q* = 87.892, *df* = 14, *p* < 0.001; *I^2^* = 84.071%). Conversely, the physically impaired group displayed no heterogeneity in the overall effect size, *r* = 0.366, *95% CI =* [0.315, 0.414], across 5 studies (*Q* = 4.171, *df* = 4, *p* = 0.383; *I^2^* = 4.098%). Notably, confidence intervals for the no-impairment and physical-impairment groups did not overlap, suggesting greater relevance of the former.

Additionally, confidence intervals for the mental-impairment and physical-impairment groups had a noticeable gap with slight overlap, implying the former’s likely greater relevance. Furthermore, neither the depression measurement tool (*Q* = 6.892, *df* = 5, *p* = 0.229) nor the alexithymia measurement tool (*Q* = 0.031, *df* = 1, *p* = 0.861) appeared to significantly contribute to heterogeneity between studies, thus Hypothesis 5 was not supported. Therefore, different measurement instruments for depression and alexithymia did not significantly influence the relationship. Information of subgroup comparisons is shown in Table 4.

**Table 4:** Results of subgroup random effects models for group types and measurement tools.

Regulating factors	Category	*k*	*N*	*r*	95% CI		*Z*	Heterogeneity test		
					Lower	Upper		Qb	df	*p*
group types	Mental disorder	15	4,352	0.470	0.393	0.540	10.58^***^	9.999	2	0.007
	Physical impairment	5	1,312	0.366	0.315	0.414	13.07^***^			
	Barrier-free	15	17,421	0.461	0.419	0.501	18.85^***^			
Depressive	BDI	10	3,306	0.431	0.359	0.498	10.51^***^	6.892	5	0.229
	BDI-II	10	2,520	0.457	0.383	0.525	10.71^***^			
	CES-D	4	2,276	0.539	0.444	0.622	9.44^***^			
	HMAD	4	407	0.447	0.292	0.579	5.23^***^			
	SDS	3	11,265	0.419	0.403	0.434	47.34^***^			
	Other	4	3,311	0.434	0.217	0.610	3.73^***^			
Aexithymia	TAS-20	33	22,879	0.457	0.418	0.494	19.95^***^	0.031	1	0.861
	TAS-26	2	206	0.438	0.209	0.622	3.57^***^			

^***^p < 0.001.

In addition, publication year and sex ratio were treated as continuous predictor variables in a meta-regression analysis to explore their potential impact on the heterogeneity of results. The results indicated a non-significant regression for sex ratio [*β* = −0.139, *SE* = 0.086, *Z* = −1.61, *p* = 0.107, *95% CI* (−0.307, 0.030)], thus failing to support Hypothesis 4. Similarly, publication year did not explain a significant portion of the variability in effect size [*β* = 0.001, *SE* = 0.003, *Z* = 0.26, *p* = 0.792, *95% CI* (−0.006, 0.007)], indicating that the association between alexithymia and depression has remained stable over time and thereby failing to support Hypothesis 6.

## 4 Discussion

### 4.1 Effects of alexithymia on depression

Alexithymia, recognized as a vulnerability for depression, is notably shaped by cross-cultural dynamics. Individuals from Eastern cultures tend to exhibit higher levels of alexithymia compared to those from Western cultures (Dion, 1996; Le et al., 2002). This discrepancy likely arises from cultural variations in attitudes towards individualism and the expression of inner feelings, rather than biological factors or differences in cognitive abilities. Rooted in Confucian principles, Chinese culture places emphasis on virtues and social obligations (Yao, 2000). Unlike Western societies, East Asian cultures prioritize emotional restraint over the exploration and outward expression of emotions (Butler et al., 2007; Dere et al., 2013). Therefore, understanding the relationship between alexithymia and depression requires careful consideration of cross-cultural factors. While previous meta-analyses have primarily focused on Western literature, our study aims to incorporate a broader range of literature from Eastern cultural backgrounds to provide a more comprehensive perspective on this relationship.

Furthermore, culture itself may act as a potential risk factor for certain mental health conditions, including depression, which can be influenced by environmental factors. Confucian societies tend to exhibit higher levels of depression indicators and lower resilience scores among adolescents compared to European societies, with significant disparities noted. These societies often uphold stricter standards, potentially contributing to heightened levels of anxiety and self-doubt among Asian students (Stankov, 2010). Existing research highlights a connection between Confucian personality traits, particularly moderate thinking, and emotional relationships in Chinese culture. The doctrine of the mean, central to Confucian ideal personality, promotes a balanced approach in behavior and interpersonal interactions, restricting emotional expression and potentially exacerbating negative emotions, thus increasing the risk of depression (Ma et al., 2015). Additionally, individuals with alexithymia experience difficulties in processing and regulating emotions, which can predispose them to psychological issues such as depression (Preece et al., 2022), as supported by numerous studies (Kenangil et al., 2020; Farhoumandi et al., 2022; Shi et al., 2022).

In the extended process theory of emotion regulation, Gross (2015) highlights that emotion identification is pivotal for adaptive emotion regulation. Individuals with alexithymia often face challenges in recognizing and articulating their feelings. Previous studies have demonstrated their struggle in regulating negative emotions or their correlation with negative emotions (De Gucht et al., 2004; Yelsma, 2007). This difficulty may consequently exacerbate depression or lead to heightened levels of depression as negative emotions accumulate.

Our study revealed varying correlations between Toronto Alexithymia Scale (TAS) sub-dimensions and depression. Difficulty Identifying Feelings (DIF) showed the strongest correlation, followed by Difficulty Describing Feelings (DDF), while Externally Oriented Thinking (EOT) exhibited the lowest correlation. This aligns with findings by De Gucht et al. (2004), who emphasized DIF as a significant predictor of depression levels. Despite EOT showing a weaker correlation, it remains relevant in understanding depression and alexithymia. Cohen (1988) warned against dismissing small effects, as they can still be meaningful. Previous research indicated that high EOT scores in depressed patients are linked to concrete thinking and poor introspection, associated with cognitive deficits and psychomotor retardation (De Berardis et al., 2008; Tominaga et al., 2014). Moreover, the reliability issue of EOT intersects with cultural differences, underscoring the need to consider linguistic and cultural contexts in assessment tools.

Our findings indicate a significant moderating effect of the tested group on the relationship between depression and alexithymia, particularly within the mental disorders group. This supports previous studies emphasizing the role of psychological factors in alexithymia (Obeid et al., 2020). Theory of Mind (ToM), or mentalizing, involves understanding one’s own and others’ mental states to predict and explain behaviors (Moriguchi et al., 2006). Individuals with high alexithymia levels often struggle with ToM, leading to difficulties in social interactions and relationships (Demers and Koven, 2015). Moreover, evidence indicates that individuals with alexithymia exhibit a limited response to psychotherapy (Ogrodniczuk et al., 2005). For instance, individuals with high levels of alexithymia on the autism spectrum often face challenges in recognizing emotions conveyed through facial expressions, vocal intonations, and musical cues (Allen and Heaton, 2010). Consequently, these individuals may have unique needs that necessitate specific interventions and could benefit from tailored therapeutic approaches (Kinnaird et al., 2019).

The study highlighted the potential interference of depression measurement tools with the effect of alexithymia on depression. This interference may arise from differences in scoring methods, target groups, assessment approaches, and scale items across various scales, leading to potential inaccuracies in information and resulting in varied final correlations. Notably, variations in total scores and prevalence of depressive symptoms among different scales may be due to their distinct focuses in assessing depressive symptoms. For example, the CES-D primarily measures depressive symptom levels within the general population over the current week, emphasizing affective components and depressive states (Radloff, 1977). In contrast, the BDI-II-C assesses the severity of depressive symptoms concerning clinical diagnosis. Additionally, depression scales often originate from foreign sources, raising concerns about equivalence across dimensions such as content, semantics, methodology, norms, and conceptualization, as noted by Flaherty et al. (1998) in cross-cultural research contexts.

Regarding gender and publication year, the study did not find significant differences in the current effect of alexithymia on depression. Previous findings on gender differences in alexithymia have varied. Levant et al. (2006) observed higher alexithymia scores among males in some studies, while others reported higher scores among females or no significant differences. Our study aligns with the latter trend. Levant et al. (2006) reviewed multiple studies and noted that while gender differences in alexithymia were generally insignificant in clinical samples, males tended to score higher in non-clinical samples. The absence of gender differences in clinical samples might be attributed to emotion recognition and expression processes in men seeking psychotherapy. Additionally, it has been suggested that the etiology or pathogenesis of alexithymia differs between genders and may be influenced by traumatic experiences, leading to variations in neuroendocrine functioning, which itself differs between males and females (Spitzer et al., 2005). Consequently, future research on gender differences in alexithymia warrants a more comprehensive approach. Conversely, the non-significance of publication year suggests that alexithymia may not only serve as a risk factor for depression but also persist as a stable personality trait (Hemming et al., 2019), resistant to change over shorter periods.

### 4.2 Limitations and implications

This study delves into the correlation between narrative affective disorder and depression, alongside its influencing factors. However, it also highlights some limitations. Firstly, the reviewed literature mainly employed the TAS scale to measure narrative affective disorder. While efficient, the TAS-20 may not accurately capture the extent of dysphoria within a sample. Individuals with heightened narrative dysphoria may struggle to reliably assess their deficits using self-report scales (Taylor and Bagby, 2013). To address this limitation, some scholars have suggested using clinician-rated instruments like the Toronto Structured Interview for Narrative Disorders (Bagby et al., 2005). However, these tools have their own constraints, including high costs, the need for interviewer training, reliance on interviewer quality, and uncertainties surrounding interview outcomes (Cameron et al., 2014). Individuals with elevated levels of dysarthria and those with Autism Spectrum Conditions (ASC) may encounter difficulties in self-awareness and articulating aspects of themselves (Griffin et al., 2016), potentially leading to underestimation of dysgraphia (and ASC). It has been proposed that the efficacy of the BVAQ-40 could be enhanced. The BVAQ-40, an alternative instrument to the TAS-20, is commonly used to evaluate the cognitive and affective dimensions of affective disorders (Goerlich and Aleman, 2018). However, the assertion regarding an affective component within narrative affective disorders remains subject to scholarly debate (Bagby et al., 2007; Preece et al., 2017; Goerlich, 2018). Therefore, future research should acknowledge this debate and explore methodologies for measuring dysarthria that do not solely rely on self-insight (Bird and Cook, 2013).

Secondly, regarding the subject group, this study noted that individuals with mental disorders exhibited the highest effect sizes. However, it overlooked the extent of dysphoria within this subgroup, potentially underestimating the significance of co-occurring dysphoria as a critical risk factor. This conclusion requires further validation through a comprehensive integration of subgroups based on their individual levels of dysphoria. Moreover, prior research indicates that socio-demographic variables such as gender, age, and education level may independently or jointly influence the association between dysphoria and depression severity (Mattila et al., 2006; Kim et al., 2009; Luca et al., 2013). These relationships warrant further exploration.

Lastly, considering that this study collected data from multiple countries within the North America-East Asia dichotomy, we employed the Individualism–Collectivism (IDV-COL) cultural dimension to examine the cultural impact on the correlations between alexithymia and depression. The IDV-COL dimensions demonstrate robust reliability and validity (Minkov et al., 2018). However, this research has limitations. While the IDV-COL framework provides valuable insights into overarching cultural influences, it does not fully capture the complexities of intra-cultural variations that can significantly shape emotional perception and expression. Specifically, cultural groups may exhibit diversity in emotional processes based on factors such as regional traditions and collective experiences (Mesquita, 2001; Mesquita and Walker, 2003; Tsai and Levenson, 1997). Consequently, the failure to account for these intra-cultural nuances may limit the generalisability of our findings.

## 5 Conclusion

Using a meta-analytic approach, this study established significant correlations between total scores of affective disorders and depression. Specifically, Difficulty Identifying Feelings (DIF) and Difficulty Describing Feelings (DDF) showed strong correlations with depression, while Externally Oriented Thinking (EOT) exhibited a weaker correlation.

The correlation between depression and dysphoria was notably influenced by the characteristics of the group under investigation and the depression measurement tool, with no apparent impact from gender or publication age. These findings contribute to the theoretical understanding of trait inference in depression and may have implications for social cognitive research in clinical disorders.

## Data Availability

The original contributions presented in the study are included in the article/Supplementary material, further inquiries can be directed to the corresponding author.
