# Supplementary material for: The effect of alexithymia on depression: evidence from meta-analysis
Source: Front Psychol. 2025 Jun 2;16:1465286. doi: 10.3389/fpsyg.2025.1465286 (PMC12171140; doi:10.3389/fpsyg.2025.1465286)
Supplement: Supplementary file 1 [file Table_1.docx]

**Supplemental Material**

**Included subgroup analyses of TAS subscales for the meta-analysis**

| No. | author | year | sub dimension | r | Sample size | correlation direction |
| --- | --- | --- | --- | --- | --- | --- |
| 1 | Saarijärvi et al. | 2001 | DDF | 0.310 | 120 | Positive |
|  |  |  | DIF | 0.450 | 120 | Positive |
|  |  |  | EOT | 0.140 | 120 | Negative |
| 2 | Honkalampi et al. | 2001 | DDF | 0.062 | 308 | Positive |
|  |  |  | DIF | 0.296 | 308 | Positive |
|  |  |  | EOT | 0.131 | 308 | Positive |
| 3 | Hintikka et al. | 2001 | DDF | 0.500 | 1888 | Positive |
|  |  |  | DIF | 0.630 | 1888 | Positive |
|  |  |  | EOT | 0.180 | 1888 | Positive |
| 4 | Lundh et al. | 2002 | DDF | 0.140 | 88 | Positive |
|  |  |  | DIF | 0.410 | 88 | Positive |
|  |  |  | EOT | 0.280 | 88 | Negative |
| 5 | De Gennaro et al. | 2004 | DDF | 0.300 | 554 | Positive |
|  |  |  | DIF | 0.514 | 554 | Positive |
|  |  |  | EOT | 0.031 | 554 | Positive |
| 6 | Eizaguirre et al. | 2004 | DDF | 0.417 | 151 | Positive |
|  |  |  | DIF | 0.514 | 151 | Positive |
|  |  |  | EOT | 0.230 | 151 | Positive |
| 7 | Ogrodniczuk et al. | 2004 | DDF | 0.280 | 33 | Positive |
|  |  |  | DIF | 0.220 | 33 | Positive |
|  |  |  | EOT | 0.050 | 33 | Negative |
| 8 | Henry et al. | 2006 | DDF | 0.170 | 121 | Positive |
|  |  |  | DIF | 0.220 | 121 | Positive |
|  |  |  | EOT | 0.080 | 121 | Positive |
| 9 | Henry | 2006 | DDF | 0.170 | 248 | Positive |
|  |  |  | DIF | 0.220 | 248 | Positive |
|  |  |  | EOT | 0.080 | 248 | Positive |
| 10 | Motan and Gençöz | 2007 | DDF | 0.140 | 145 | Positive |
|  |  |  | DIF | 0.300 | 145 | Positive |
|  |  |  | EOT | 0.050 | 145 | Positive |
| 11 | De Berardis et al. | 2008 | DDF | 0.440 | 145 | Positive |
|  |  |  | DIF | 0.340 | 145 | Positive |
|  |  |  | EOT | 0.200 | 145 | Positive |
| 12 | Bamonti et al. | 2010 | DDF | 0.440 | 134 | Positive |
|  |  |  | DIF | 0.470 | 134 | Positive |
|  |  |  | EOT | 0.030 | 134 | Positive |
| 13 | Reker et al. | 2010 | DDF | 0.350 | 33 | Positive |
|  |  |  | DIF | 0.360 | 33 | Positive |
|  |  |  | EOT | 0.330 | 33 | Negative |
| 14 | Herbert et al. | 2011 | DDF | 0.380 | 155 | Positive |
|  |  |  | DIF | 0.290 | 155 | Positive |
|  |  |  | EOT | 0.180 | 155 | Positive |
| 15 | Gilbert et al. | 2012 | DDF | 0.600 | 185 | Positive |
|  |  |  | DIF | 0.540 | 185 | Positive |
|  |  |  | EOT | 0.250 | 185 | Positive |
| 16 | Luca et al. | 2013 | DDF | 0.190 | 75 | Positive |
|  |  |  | DIF | 0.250 | 75 | Positive |
|  |  |  | EOT | 0.230 | 75 | Positive |
| 17 | Katsifaraki and Tucker | 2013 | DDF | 0.310 | 183 | Positive |
|  |  |  | DIF | 0.330 | 183 | Positive |
|  |  |  | EOT | 0.120 | 183 | Positive |
| 18 | Craparo et al. | 2014 | DDF | 0.327 | 80 | Positive |
|  |  |  | DIF | 0.352 | 80 | Positive |
|  |  |  | EOT | 0.227 | 80 | Positive |
| 19 | Yalinay Dikmenet al. | 2020 | DDF | 0.451 | 145 | Positive |
|  |  |  | DIF | 0.473 | 145 | Positive |
|  |  |  | EOT | 0.302 | 145 | Positive |
| 20 | Tan et al. | 2021 | DDF | 0.370 | 365 | Positive |
|  |  |  | DIF | 0.430 | 365 | Positive |
|  |  |  | EOT | 0.150 | 365 | Positive |
| 21 | Pei et al | 2021 | DDF | 0.386 | 413 | Positive |
|  |  |  | DIF | 0.486 | 413 | Positive |
|  |  |  | EOT | 0.309 | 413 | Positive |
| 22 | Tang et al. | 2022 | DDF | 0.400 | 1270 | Positive |
|  |  |  | DIF | 0.530 | 1270 | Positive |
|  |  |  | EOT | 0.120 | 1270 | Positive |
| 23 | Oakley 1 et al. | 2022 | DDF | 0.490 | 337 | Positive |
|  |  |  | DIF | 0.640 | 337 | Positive |
|  |  |  | EOT | 0.080 | 337 | Positive |
| 24 | Oakley 2 et al. | 2022 | DDF | 0.310 | 179 | Positive |
|  |  |  | DIF | 0.590 | 179 | Positive |
|  |  |  | EOT | 0.030 | 179 | Positive |
| 25 | Oakley 3 et al. | 2022 | DDF | 0.300 | 135 | Positive |
|  |  |  | DIF | 0.420 | 135 | Positive |
|  |  |  | EOT | 0.090 | 135 | Positive |
| 26 | Oakley 4 et al. | 2022 | DDF | 0.200 | 76 | Positive |
|  |  |  | DIF | 0.300 | 76 | Positive |
|  |  |  | EOT | 0.020 | 76 | Negative |
| 27 | Kenangil et al. | 2023 | DDF | 0.200 | 42 | Positive |
|  |  |  | DIF | 0.320 | 42 | Positive |
|  |  |  | EOT | 0.130 | 42 | Negative |
| 28 | Ozonder Unal and Ordu | 2023 | DDF | 0.230 | 151 | Positive |
|  |  |  | DIF | 0.198 | 151 | Positive |
|  |  |  | EOT | 0.246 | 151 | Positive |
| 29 | lv 1 et al. | 2023 | DDF | 0.404 | 40 | Positive |
|  |  |  | DIF | 0.321 | 40 | Positive |
|  |  |  | EOT | 0.056 | 40 | Positive |
| 30 | lv 2 et al. | 2023 | DDF | 0.379 | 86 | Positive |
|  |  |  | DIF | 0.332 | 86 | Positive |
|  |  |  | EOT | 0.008 | 86 | Negative |
